# Impact of the Sub-Resting Membrane Potential on Accurate Inference in Spiking Neural Networks

**DOI:** 10.1038/s41598-020-60572-8

**Published:** 2020-02-26

**Authors:** Sungmin Hwang, Jeesoo Chang, Min-Hye Oh, Jong-Ho Lee, Byung-Gook Park

**Affiliations:** Inter-university Semiconductor Research Center (ISRC) and Department of Electrical and Computer Engineering, Seoul National University, Seoul, 08826 Republic of Korea

**Keywords:** Electrical and electronic engineering, Computer science

## Abstract

Spiking neural networks (SNNs) are considered as the third generation of artificial neural networks, having the potential to improve the energy efficiency of conventional computing systems. Although the firing rate of a spiking neuron is an approximation of rectified linear unit (ReLU) activation in an analog-valued neural network (ANN), there remain many challenges to be overcome owing to differences in operation between ANNs and SNNs. Unlike actual biological and biophysical processes, various hardware implementations of neurons and SNNs do not allow the membrane potential to fall below the resting potential—in other words, neurons must allow the sub-resting membrane potential. Because there occur an excitatory post-synaptic potential (EPSP) as well as an inhibitory post-synaptic potential (IPSP), negatively valued synaptic weights in SNNs induce the sub-resting membrane potential at some time point. If a membrane is not allowed to hold the sub-resting potential, errors will accumulate over time, resulting in inaccurate inference operations. This phenomenon is not observed in ANNs given their use of only spatial synaptic integration, but it can cause serious performance degradation in SNNs. In this paper, we demonstrate the impact of the sub-resting membrane potential on accurate inference operations in SNNs. Moreover, several important considerations for a hardware SNN that can maintain the sub-resting membrane potential are discussed. All of the results in this paper indicate that it is essential for neurons to allow the sub-resting membrane potential in order to realize high-performance SNNs.

## Introduction

**Figure 1:**
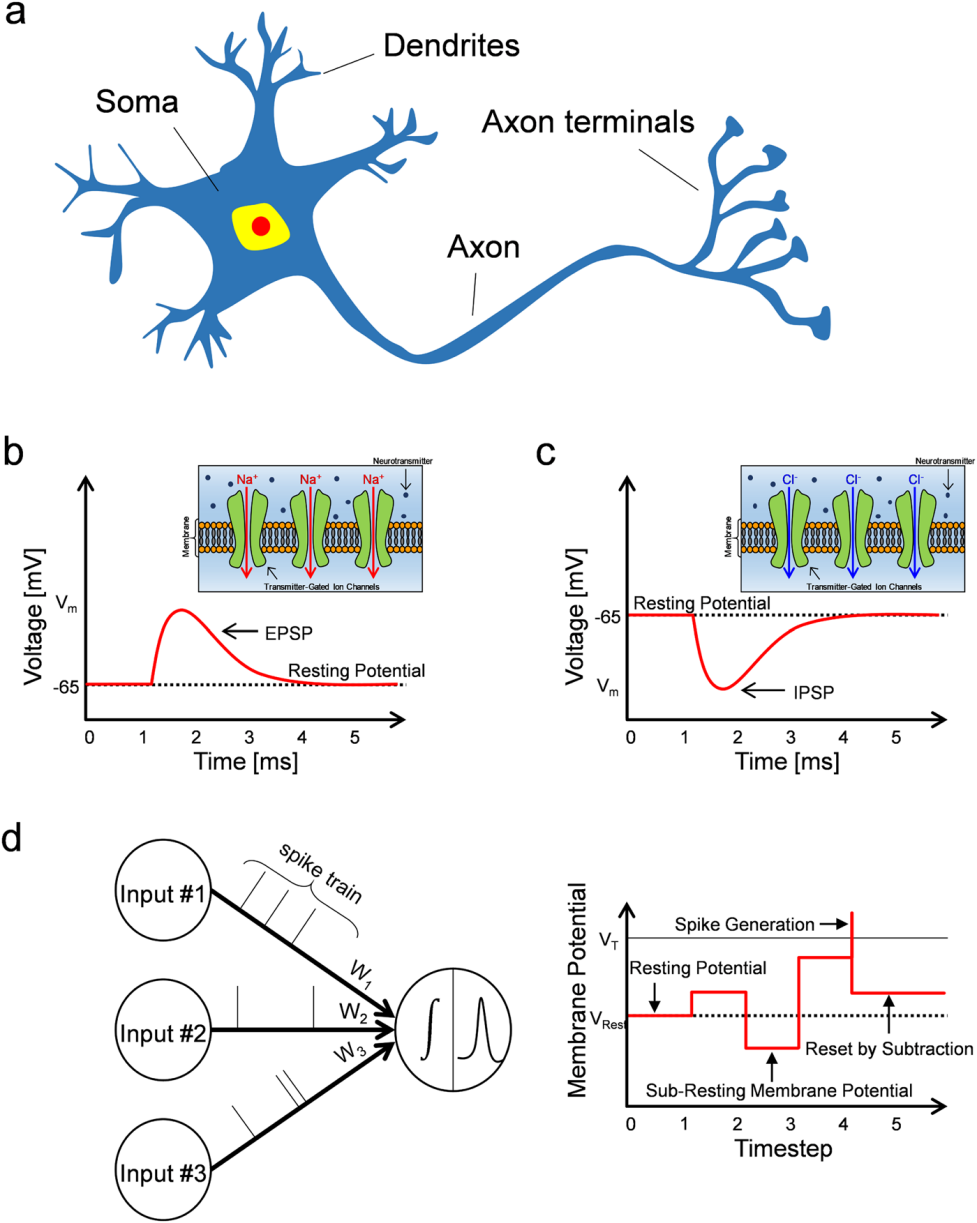
(**a**) Illustration of a biological neuron. A neuron receives pre-synaptic stimulus from dendrites, and synaptic integration occurs in soma. When a membrane voltage exceeds a threshold, a neuron generates an action potential, which is transmitted to other neurons through axon. (**b,c**) Neurotransmitter in synaptic vesicle is released by an action potential. Stimulation of transmitter-gated ion channels permeable to Na^+^ and Cl^-^ induces EPSP (depolarization) and IPSP (hyperpolarization), respectively. It is a biologically plausible to allow the sub-resting membrane potential from IPSP characteristic. (**d**) Schematic representation of spiking neural networks (SNNs). Input signals are encoded by spike train, and temporal and spatial integration of inputs multiplied by synaptic weights occur. During temporal integration, the sub-resting membrane potential can be momentarily induced when negatively valued weighted input sum dominates.

Spiking neural network (SNN) has the potential to change the conventional computing paradigm, in which analog-valued neural network (ANN) is currently predominant^1,2^. ANNs and SNNs are analogous in that they consist of neurons and synapses connected in a massively parallel fashion, but SNNs are based on more biologically plausible neuron models where a signal is propagated in the form of a spike. Like a biological nervous system, therefore, the SNN is an event-driven system that performs computations only when a spike occurs. Accordingly, SNNs are considered to be suitable for an energy-efficient computing system^3–6^. Numerous studies have attempted to implement various ANN applications in SNN manner^7–10^, but there remain a number of challenges to be resolved in order to utilize SNNs with practical applications. Rueckauer *et al*. proposed a neuron model with what is termed a ‘reset by subtraction’ operation, during which the membrane potential is reset by subtracting the amount of the threshold to prevent a loss of information, which causes a reduction of the firing rate^8^. An additional issue to be addressed is that the membrane potential of neurons in SNNs must be allowed to fall below the resting potential—in other words, neurons must allow the sub-resting membrane potential. Most software implementations of SNNs do not limit neural membrane potentials by stipulating that they must remain above the resting potential^11–13^, but there are a few exceptions^14^. Cao *et al*. noted that the minimum value of the membrane potential is allowed to be the resting potential or should be lower than this level^15^; however, the theoretical background behind this concept was lacking, and no study has analyzed the impact when the lower bound of the membrane potential is limited to the resting potential. Specifically, given that a number of hardware implementations of spiking neurons and SNNs tend to restrict the membrane potential from going below the resting potential, it is important explicitly to investigate the impact of the sub-resting membrane potential on SNNs^16–22^. Biological neurons consist of dendrites, the soma, and axons, as shown in Fig. 1a. Dendrites receive signals from pre-synaptic neurons and spatio-temporally integrate them into the soma. When the accumulated potential in the soma exceeds the neuron’s threshold, the neuron generates a spike, which is transmitted along the axon to the next neuron^23,24^. The signal from a pre-synaptic neuron can increase or decrease a membrane potential of the soma. For example, if neurotransmitters such as acetylcholine (ACh) and glutamate (Glu) released by a pre-synaptic signal stimulate ion channels which are permeable to Na^+^ at the neuromuscular junction, this action will depolarize the post-synaptic cell, causing the membrane potential to move toward the threshold. Transient depolarization is also known as the excitatory post-synaptic potential (EPSP), as illustrated in Fig. 1b. In contrast, if the ion channels stimulated by neurotransmitters, such as gamma-aminobutyric acid (GABA) and glycine (Gly), are permeable to Cl^−^, this action will hyperpolarize the membrane potential from the resting potential. Transient hyperpolarization of the membrane potential is termed the inhibitory post-synaptic potential (IPSP), as illustrated in Fig. 1c^23–25^. Therefore, it is plausible on the basis of neurobiological principles that neurons in SNNs must allow the sub-resting membrane potential. Specifically, there are negatively valued weights as well as positively valued weights in neural networks; hence, input spikes connected with negatively valued synaptic weights can cause the sub-resting membrane potential during temporal synaptic integration, as illustrated in Fig. 1d. This phenomenon is not observed in ANNs because there is only spatial synaptic integration, but it can cause serious performance degradation in SNNs. If the membrane potential has a lower bound, the amount that cannot fall below the resting potential induces errors over time.

This is a fundamental issue in relation to SNNs. In particular, its impact has worsened as numerous neural network applications are composed of multiple hidden layers. In this work, we analyze the impact of the sub-resting membrane potential on the accurate inference of SNNs. We employ integrate-and-fire neurons (I&F neurons) where one has the lower bound of the membrane potential as the resting potential and the other is capable of retaining the sub-resting membrane potential. By comparing the inference performance when each neuron is applied to SNN applications involving MNIST and CIFAR-10 classifications and an autoencoder, the validity of allowing the sub-resting membrane potential for SNN neurons is demonstrated. Moreover, considerations of interest for implementing a hardware SNN that can hold the sub-resting membrane potential are discussed. In the following sections, we set the resting potential to zero for ease of implementation and simplicity. Hence, the sub-resting membrane potential is referred as the negative membrane potential (NMP). All simulations are conducted using *PyTorch ver*. 1.0.0.

## Theory

In order to model the impact of a negative membrane potential (i.e. sub-resting membrane potential), we start with a simple integrate-and-fire neuron model based on previous work^8^. The spike generation of *i*-th neuron in layer *l* at time *t* can be represented as:

1 $${\delta }_{i}^{l}(t)=\{\begin{array}{c}1,when\,there\,is\,a\,spike\\ 0,\,otherwise\,\end{array}$$

where *δ*^0^ is the input.

The sum of the input integrated at the membrane of the *i*-th neuron in layer *l* at time *t* is defined as:

2 $${z}_{i}^{l}(t)={\sum }_{j=1}^{{M}^{l-1}}{w}_{ij}^{l}{\delta }_{j}^{l-1}(t)+{b}_{i}^{l}$$

where *M*^*l*−1^ is the number of neurons in layer *l−*1 and ${b}_{i}^{l}$ is the bias.

${z}_{i}^{l}(t)$ becomes negative when the negatively weighted input sum is dominant over the positively weighted input sum, which can induce a negative membrane potential. We can define the membrane potential of the *i*-th neuron in layer *l* at time *t* as:

3 $${V}_{i}^{l}(t)={V}_{i}^{l}(t-1)+{z}_{i}^{l}(t)-{V}_{th}{\delta }_{i}^{l}(t)+{{\epsilon }}_{i}^{l}(t,{v}_{lb})$$

where ${{\epsilon }}_{i}^{l}(t,\,{v}_{lb})$ is the error term generated by the lower bound of the membrane potential $\,{v}_{lb}$. The error term ${{\epsilon }}_{i}^{l}(t,\,{v}_{lb})$ is expressed as follows:

4 $${{\epsilon }}_{i}^{l}(t,{v}_{lb})=\{\begin{array}{c}0,when\,{v}_{lb}=-\infty \\ {{\epsilon }}_{i}^{l}(t,a)\ge {{\epsilon }}_{i}^{l}(t,b)\ge 0\,for\,all\,a\ge b\ge -\infty \end{array}$$

when the lower bound of the membrane potential is restricted to zero, the error *ϵ* can occur during the temporal integration process. Summing over and dividing by the total simulation time *t* for layer $l=1$, we obtain:

5 $$\frac{1}{t}{\sum }_{t{\prime} =1}^{t}{V}_{i}^{1}(t{\prime} )=\frac{1}{t}{\sum }_{t{\prime} =1}^{t}\{{V}_{i}^{1}(t{\prime} -1)+{z}_{i}^{1}(t{\prime} )-{V}_{th}{\delta }_{i}^{1}(t{\prime} )+{{\epsilon }}_{i}^{1}(t{\prime} ,{v}_{lb})\}$$

Assuming *V*_th_ = 1, the average firing rate ${r}_{i}^{l}(t)$ of the *i*-th neuron in layer $l=1$ at time *t* can be defined as:

6 $${r}_{i}^{1}(t)=\frac{{N}_{i}^{1}(t)}{t}=\frac{1}{t}{\sum }_{t{\prime} =1}^{t}{z}_{i}^{1}(t{\prime} )-\frac{{V}_{i}^{1}(t)-{V}_{i}^{1}(0)}{t}+\frac{1}{t}{\sum }_{t{\prime} =1}^{t}{{\epsilon }}_{i}^{1}(t{\prime} ,\,{v}_{lb})={f}_{i}^{1}(t)+{E}_{i}^{1}(t)$$

where ${N}_{i}^{l}(t)$ is the total number of spikes generated in the *i*-th neuron in layer *l* during time *t*.

That is, if we do not allow the negative membrane potential, neurons tend to fire more (${E}_{i}^{1}(t)$) than the correct number of spikes (${f}_{i}^{1}(t)$). This becomes worse in deeper neural networks. By recursively calculating Eqs. (1) through (6) in layer $l$, we can determine the average firing rate ${r}_{i}^{l}$ as shown below.

7 $${r}_{i}^{l}(t)={\sum }_{j=1}^{{M}^{l-1}}{w}_{ij}^{l}{f}_{i}^{l-1}(t)+{r}_{max}\cdot {b}_{i}^{l}-\frac{{V}_{i}^{l}(t)-{V}_{i}^{l}(0)}{t}\,+\,{\sum }_{j=1}^{{M}^{l-1}}{w}_{ij}^{l}{E}_{i}^{l-1}(t)+{E}_{i}^{l}(t)\,$$

Here, *r*_*max*_ is the maximum firing rate that a neuron can generate. It is defined as *r*_*max*_ = 1/Δ*t*, where Δ*t* is the minimum timestep.

The new error caused by limiting the membrane potential to zero, the fifth term in Eq. (7), is added to the inputs which were fired incorrectly due to the error in the previous layer, i.e., the fourth term in Eq. (7). The fifth term indicates that the time-averaged error becomes a non-negative constant as the network reaches a steady state. The fourth term reflects the accumulation of errors through layer-to-layer propagation and can be positive or negative depending on the dominant weights. Consequently, even after a long inference operation, the error caused by the lower bound of the membrane potential persists and accumulates as it propagates through the layers.

## Results

Two major reasons why ANNs have achieved great success are their excellent learning algorithms (error-backpropagation) and the rectified-linear unit (ReLU) activation function which enables deep layers to be trained without the vanishing gradient problem^26^. However, it is difficult to train SNNs using the same learning algorithm due to differences in signal forms^27–29^. Although biological learning algorithms (e.g., spike-timing-dependent plasticity (STDP) and spike-rate-dependent plasticity (SRDP)) have been widely used to train SNNs, only a few studies have achieved a level of performance comparable to that by ANNs^30–33^. In recent studies, many research groups have suggested ANN-to-SNN conversion methods that implement the SNN inference system by mapping the weights trained in ANNs^7–9^. This is possible because the output firing rate of an I&F neuron in a SNN is an approximation of the output activation of ReLU in an ANN. Therefore, the time-integration of output spikes can represent values equivalent to ANN activations^7–9,15^. In this work, we demonstrate the effect of a negative membrane potential using ANN-to-SNN conversion methods, as these demonstrate performance closest to that of an ANN. Nevertheless, the effect discussed later is not limited to SNNs implemented in any particular manner because it is a solution to the fundamental problem of SNNs.

### Inference performance

**Figure 2:**
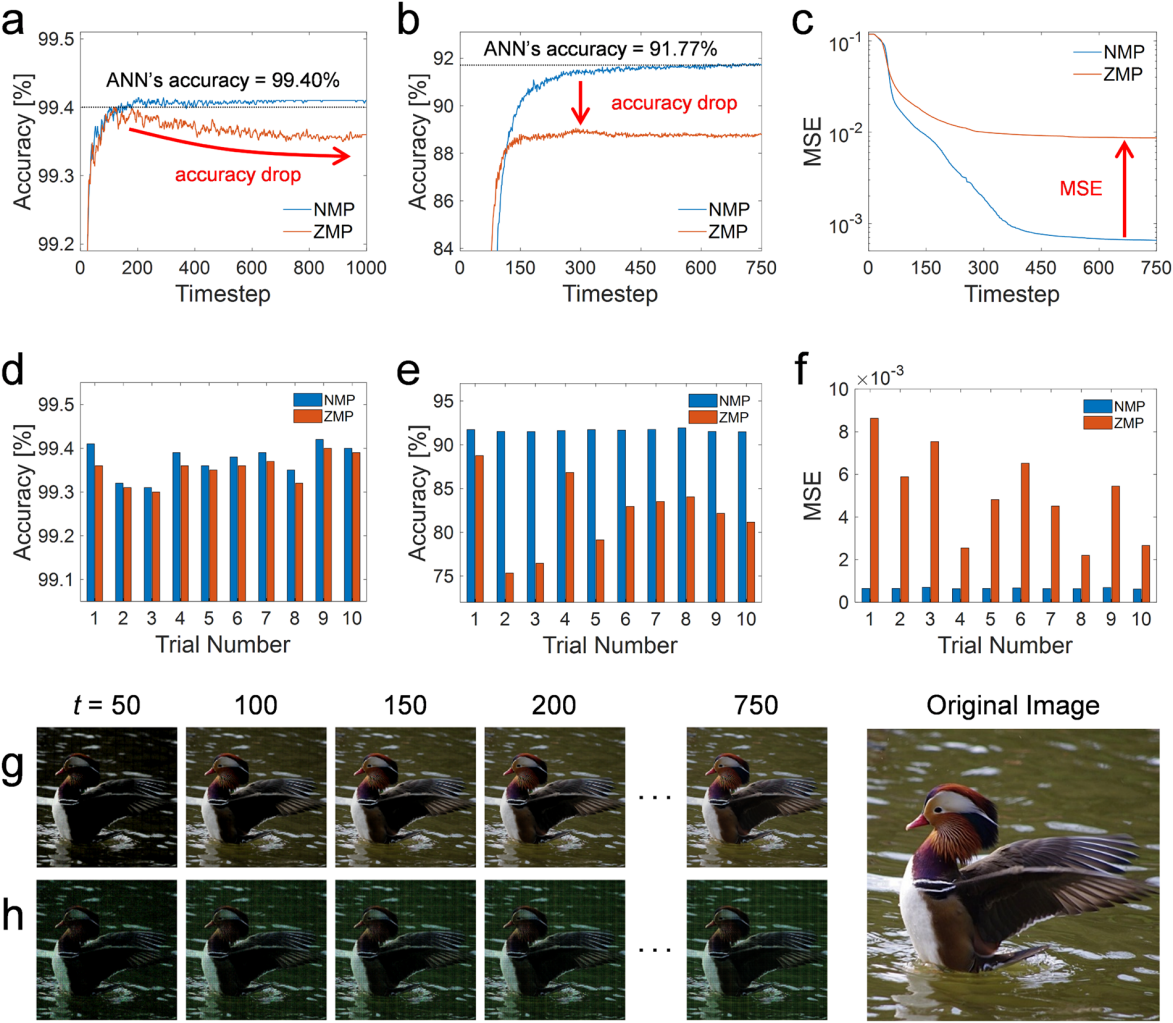
Simulation results according to the simulation timestep. (**a**) In MNIST classification problem, it seems that there is no difference between the NMP and ZMP cases. However, the accuracy gradually decreases in the ZMP case with long simulation time. (**b**) In CIFAR-10 classification case, there is a large accuracy drop with the ZMP case compared with the result of the NMP case. (**c**) For spiking autoencoder, MSE decreases according to the simulation time in both the NMP and ZMP cases, but a large difference occurs. (**d**–**f**) Changes of the classification accuracy and MSE with 10 random seeds for weight initialization extracted at a steady-state. The performance of the NMP case is stable and higher than that of the ZMP case at all the trials. (**g**,**h**) Changes of a sample image reconstructed by autoencoder at 50^th^, 100^th^, 150^th^, 200^th^, and 750^th^ timesteps for the NMP and ZMP cases. The reconstructed image for the NMP case is very close to the original image, but the image for the ZMP case is not restored.

Figure 2a shows the classification accuracy of the SNN according to the simulation time for the MNIST dataset where the blue and orange lines correspond to cases with the negative membrane potential (NMP) and the zero-lower-bound membrane potential (ZMP), respectively. The classification accuracy of the SNN reaches 99% of the ANN’s accuracy (98.41%) within 20 timesteps for both the NMP and ZMP cases. It appears that there is no difference between the NMP and ZMP cases, as the accuracy rates in both cases approach that of the ANN within a short time. When the inference runs for a longer time (up to the 1,000^th^ timestep in this work), however, the accuracy of the ZMP decreases gradually to 99.36% which is below the best accuracy of the ZMP (99.42%). On the other hand, for the NMP case, there was only a slight fluctuation around the best accuracy of the NMP (99.43%), stemming from the change in the precision of the spike rate with the simulation time^8^.

NMP can have a greater impact on deeper and more complex networks. The changes of the classification accuracy with the simulation time in the SNN with CIFAR-10 for the NMP and ZMP cases are illustrated in Fig. 2b. In the NMP case, the accuracy reaches 99% of the ANN’s accuracy (90.85%) at the 180^th^ timestep, and the best accuracy (91.78%) exceeds that of the ANN (91.77%) at the 743^rd^ timestep. In contrast, in the ZMP case, the accuracy falls considerably by approximately 2.72%p compared to the NMP case.

Unlike classification problems in which the most frequently firing neuron of the output layer matters, SNN inference without NMP can give rise to more serious problems in applications such as an autoencoder, where the activation itself has an important meaning. The mean-square error (MSE) according to the simulation time is illustrated in Fig. 2c for the NMP and ZMP cases using a sample 512 × 512 image. Even after a sufficient simulation time (750^th^ timestep), the MSE in the ZMP case is one order of magnitude lower than that in the NMP case. The changes of a sample reconstructed image at the 50^th^, 100^th^, 150^th^, 200^th^, and 750^th^ timesteps for the NMP and ZMP cases are shown in Fig. 2g,h, respectively. For the NMP case, it takes time to converge to the original image due to the latency of the SNN, but the output becomes clear at the 750^th^ timestep, as illustrated in Fig. 2g. On the other hand, for the ZMP case, the original image is not restored even at the 750^th^ timestep, as shown in Fig. 2h.

We trained the networks with ten random seeds for weight initialization and extracted the classification accuracy and MSE for each trial when the performance converges to a steady state. For the MNIST dataset, as shown in Fig. 2d, there is little difference, but the accuracy of the NMP is slightly higher than that of the NMP in all trials. For the classification and autoencoder tasks using the CIFAR-10 dataset, as shown in Fig. 2e,f, respectively, a large performance drop is observed with the ZMP in all trials. While the accuracy and MSE of ZMP tend to greatly vary with each trial, those of the NMP remains stable continually.

Consequently, it is difficult to perform an accurate inference operation in SNNs when the NMP is not allowed, and the NMP has a clear impact on the characteristics of SNNs regardless of the initial weights during the training stage.

### Correlation diagrams

**Figure 3:**
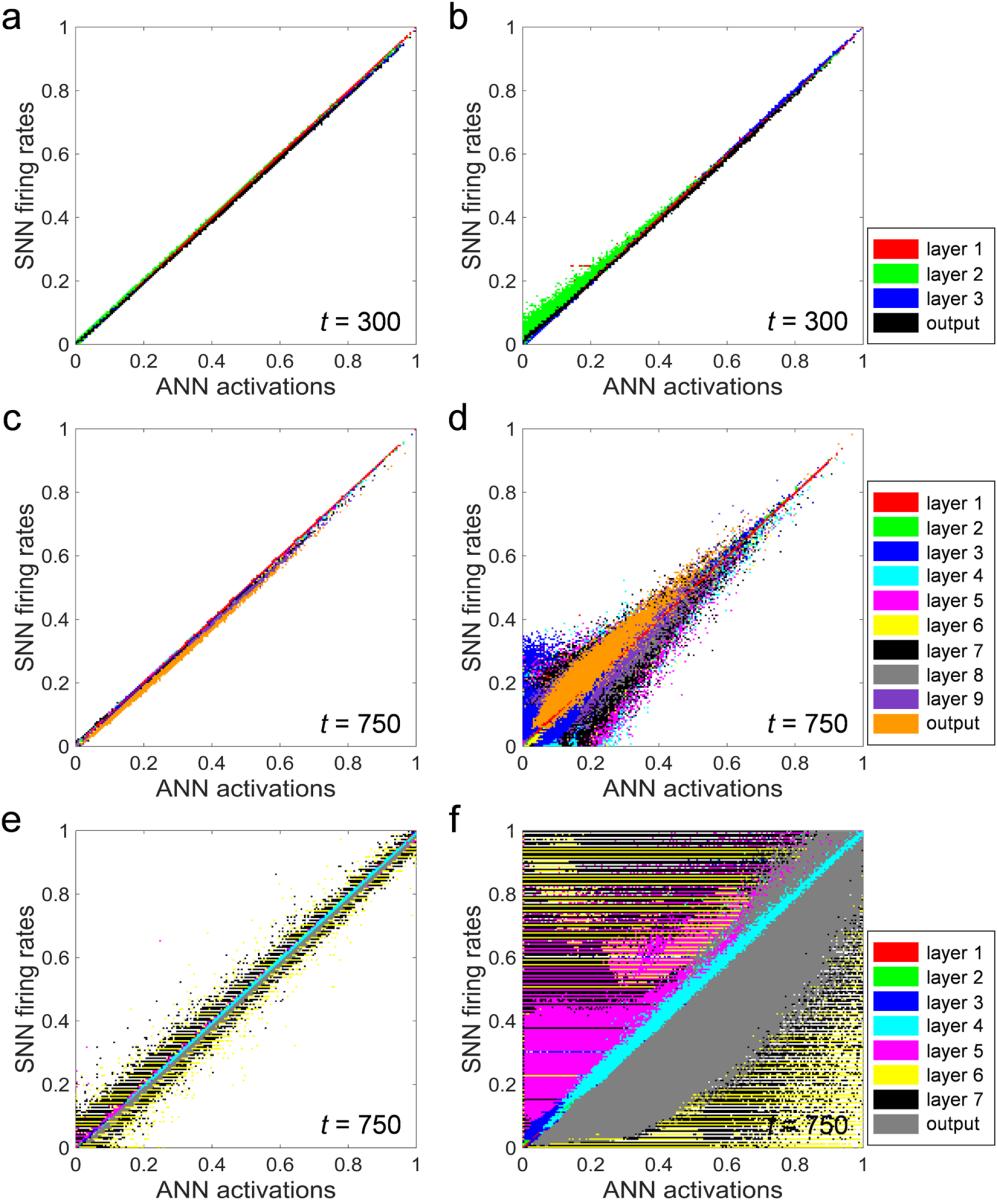
Correlation diagrams of SNN at the last simulation timestep. (**a**,**c**,**e**) The firing rates of SNN are well correlated with ANN activations for the ZMP cases of the MNIST, CIFAR-10 classifiers and autoencoder, respectively. (**b**,**d**,**f**) SNNs cannot accurately represent the ANN activations in that there is a large variation for the ZMP cases of the MNIST, CIFAR-10 classifiers and autoencoder, respectively. That is the cause of the performance drop.

In order to demonstrate the effect of the NMP in detail, the correlations between the ANN activations and the firing rates of the SNN at the 300^th^ timestep for the NMP and ZMP cases in Fig. 2a are shown in Fig. 3a,b, respectively. The correlation diagram indicates how accurately the firing rates of the SNN reproduce the ANN activations. If all points in the correlation diagram are on line *y* = *x*, the SNN firing rate is considered to match the ANN activation perfectly for all neurons. The firing rates of the SNN from all neurons in the network for 10,000 test samples are extracted by normalizing the total number of spikes with the simulation time, and they are plotted as a function of the ANN activations of the corresponding neurons. As shown in Fig. 3a, when the NMP is applied, the firing rates of the SNN are in good agreement with the ANN activations; however, several neurons fire more actively than the corresponding ANN activations in the ZMP case, causing the accuracy to drop, as shown in Fig. 3b.

For the same network in Fig. 2b using the CIFAR-10 dataset, the firing rates of all neurons in the network are consistent with the ANN activations in the NMP case, as illustrated in Fig. 3c. On the other hand, in the ZMP case, there are large deviations between the firing rates of the SNN and ANN activations in all layers, as illustrated in Fig. 3d. Unlike the correlation diagram of the network using the MNIST dataset, these deviations are due to both over- and under-fired neurons compared to the corresponding ANN neurons. Initially, the error by the ZMP induces mostly over-firing for neurons close to the input layer; however, the over-fired spikes propagate to the subsequent layers as inputs such that they lead to under-firing as well as over-firing depending on the weighted sum. The accuracy drop can be significantly affected by a large deviation in this case. This reveals that the impact of not allowing the NMP is much stronger in deeper and more complex networks.

Figure 3e,f show correlation plots of the same SNN autoencoder depicted in Fig. 2c for the NMP and ZMP cases at the 750^th^ timestep, respectively. Although a perfect correlation comparable to the classification case is not observed, the firing rates of the SNN autoencoder correspond well with the ANN activations to some extent for the NMP case, as shown in Fig. 3e. Some deviation remains, but it can be reduced, resulting in a line with a slope of 1 when the inference process involves a longer simulation time. As indicated in Fig. 3f, however, the number of neurons whose firing rate does not coincide with the ANN activation is dramatically increased and the autoencoder does not work at all in the ZMP case.

### Hardware configurations

**Figure 4:**
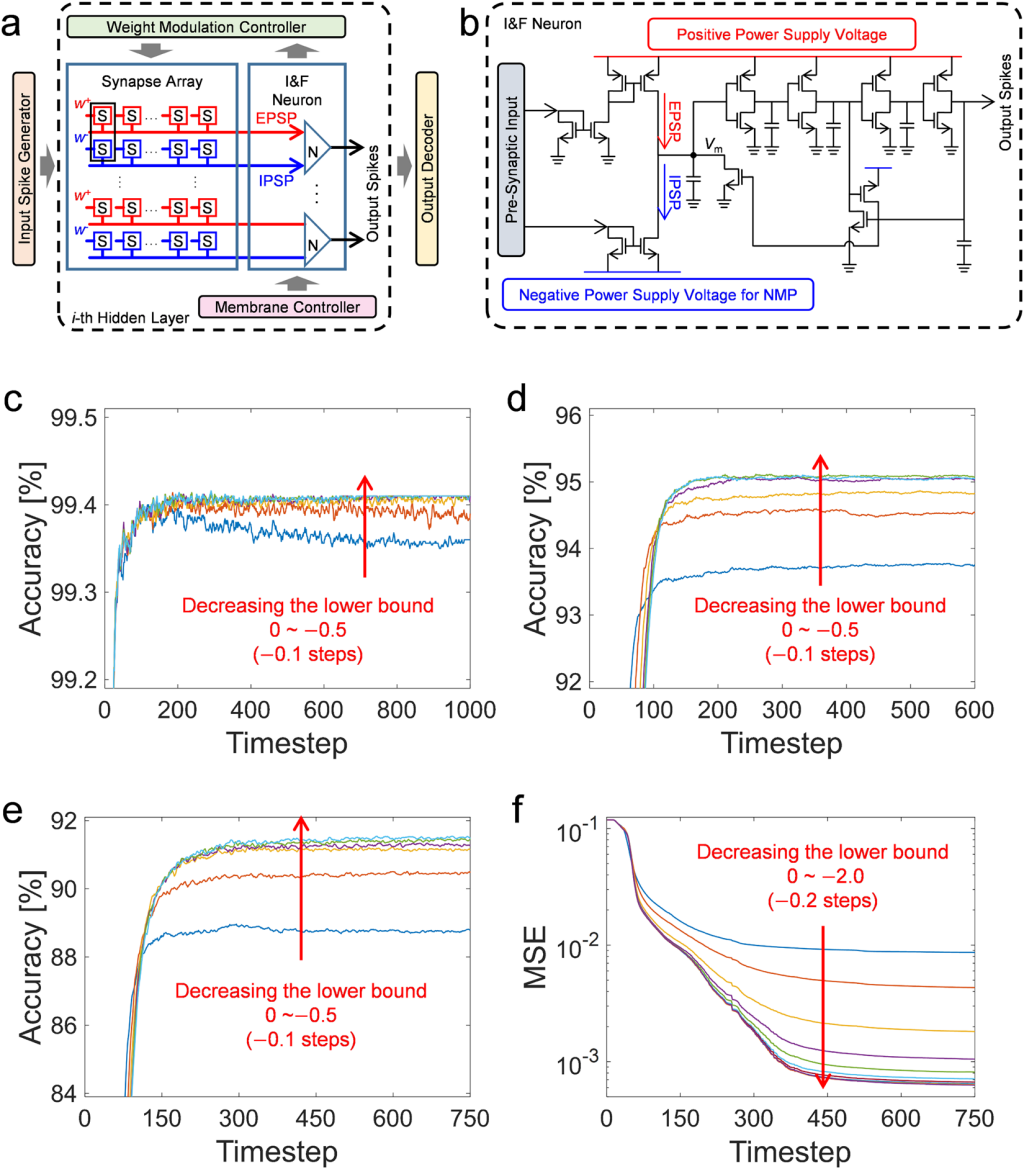
(**a**) Schematic of hardware SNN system configuration. Inputs are encoded by input spike generator, and outputs are decoded by output decoder. Hidden layers are composed of synapse and I&F neuron arrays, which are controlled by weight modulation controller and membrane controller for accurate weight transfer and membrane reset, respectively. (**b**) Circuit diagram of I&F neuron for hardware implementation of SNN. The membrane potential (*V*_m_) can retain a negative value due to the negative power supply voltage connected to the current mirror for the inhibitory synapses. (**c**–**f**) Finding the optimal lower bound of the membrane potential. The simulations are conducted with varying the lower bound of the membrane potential for the MNIST, SVHN, and CIFAR-10 classifiers and autoencoder using the test data. The converged accuracy and MSE are improved as the lower bound of the membrane potential decreases.

Operating SNNs on a conventional computing system is slower and less energy efficient due to the parameter expansion caused by neuron models as compared to operating ANNs. Thus, SNNs have advantages in terms of energy efficiency when implemented in hardware due to their event-driven processes^3–6^. Figure 4a shows an example of a hardware SNN system configuration. The input spike generator corresponds to the input layer in ANNs, composed of integrate-and-fire neurons, converting current or voltage signals from outside devices, such as image sensors, to time-series spikes whose firing rate is proportional to the amplitude of the signals. The input spikes are transferred to the *i*-th hidden layer, which consists of a neuron array, a synapse array, a weight-modulation controller, and a membrane controller. In the synapse array, a pair of synaptic devices represents one synaptic weight $({\boldsymbol{w}}={{\boldsymbol{w}}}^{+}-{{\boldsymbol{w}}}^{-})$, with which inducing the EPSP and IPSP^34,35^. The weight-modulation controller is responsible for precisely adjusting the weight of the synaptic device in consideration of hardware variations. In order to do this, the controller must monitor the firing rate of each neuron induced by a single weight and modulate its weight based on the firing information. Upon the arrival of new input data, existing information remains in the membrane, which affects the inference accuracy. Therefore, it is necessary to initialize the membrane potential in all neurons when a new pattern is applied using the membrane controller.

As shown in Fig. 4b, we propose an I&F neuron circuit which differs from the conventional I&F neuron circuit proposed earlier by the authors in that the newly proposed circuit can maintain the NMP^36,37^. Generally, CMOS I&F neurons are composed of current mirrors, a capacitor, and cascaded inverters. Pre-synaptic inputs are integrated in the capacitor through the current mirrors, and the neuron fires when the membrane potential (*V*_m_) exceeds the switching voltage of the inverter. It is possible to maintain the NMP by applying a negative power supply to the source of an n-type MOSFET consisting of a current mirror connected to inhibitory synapses. The amplitude of the negative voltage determines the lower bound of the NMP. This may not be a problem for software-based SNNs unless the lower bound of the NMP is out of the range covered by a 32-bit floating-point number; however, it is physically impossible to implement a negative supply voltage whose magnitude is infinitely large. Accordingly, one important parameter to be examined carefully during the hardware implementation of a SNN is the lower bound of the NMP. Figure 4c,d, and 4e show the changes of the classification accuracy according to the lower bound of NMP when using the MNIST, SVHN, and CIFAR-10 datasets, respectively, and Fig. 4f indicates the changes of MSE in the autoencoder using the test data with respect to the lower bound of the NMP. In Fig. 4c,d, and 4e, the converged accuracy gradually increases when the lower bound of the NMP varies from 0.0 to –0.5 in –0.1 steps. Likewise, in the case of the autoencoder, the MSE gradually decreases when the lower bound of the NMP decreases from 0.0 to –2.0 in –0.2 steps, as shown in Fig. 4f. These results indicate that the lower bound of the NMP can be optimized at different values depending on the dataset, network structure, hyper-parameter, and similarly influential parameters, also indicating, however, that the optimal lower bound of the membrane potential is not a significantly negative value. To have some margin, we propose –2 times the threshold as the lower bound of the membrane potential for hardware implementation.

First, when the negative membrane potential is not allowed, neurons having a weighted sum close to zero are most affected because the membrane potential of the neurons is in a dynamic state that instantaneously goes back and forth between a positive and negative value. In addition, when training a neural network, regularization techniques are typically used so as to improve the generalization performance. They optimize the weight values in a direction that decreases as training progresses. ANN-to-SNN conversion methods also have a weight-normalization process that considers the balance between the threshold and the weight^7,8^. With a well-tempered weight distribution, therefore, the lower bound of the membrane potential need not be a large negative value. Finally, in terms of hardware design, a circuit generating a negative supply voltage is not only difficult to implement due to its complexity but also requires extra overhead in terms of its area and energy consumption^38–41^. If the lower bound of the membrane potential is set to a small negative value, the error ${{\epsilon }}_{i}^{l}(t,{v}_{lb})$ in Eq. (3) increases. In contrast, if the lower bound of the membrane potential is set to a large negative value, the error may decrease slightly, but the energy consumption of the system increases in proportion to the square of the supply voltage ${V}_{{\rm{DD}}}^{2}$. Summing up all of these points, it appears that that −*V*_DD_ (approximately –2 times the threshold) can be an appropriate value as the lower bound of the membrane potential.

## Conclusion

In this paper, we analyzed the impact of a negative membrane potential (NMP) on accurate inference in spiking neural networks. Allowing the NMP during synaptic integration is derived from the principles of biological nervous systems, where the membrane potential is controlled by the EPSP and IPSP. The validity of allowing the NMP is verified through SNN simulations with classification and an autoencoder, the most commonly used neural network applications. In a network that recognizes relatively simple patterns (e.g., MNIST), there is little impact of not allowing the NMP; however, a large performance degradation occurs in deep and complex networks when the NMP is not allowed. The performance is degraded according to a comparison of SNN firing rates with ANN activations through correlation diagrams. We also investigated the lower bound of the NMP in relation to maintaining high performance levels during inference operations, as this must be considered during the hardware design process. All of the results here indicate that allowing the NMP is indispensable to realize an SNN inference system capable of high performance.

## Methods

### MNIST dataset

A convolutional neural network (CNN) was trained using the MNIST dataset. The CNN architecture is denoted as 20**C**3-50**C**5-**FC**500-**FC**10, where *n***C***m* indicates *n* filters of size *m* × *m* and **FC***m* denotes a fully connected layer with *m* neurons. For all hidden layers, several nodes randomly dropped out with a probability of 0.5 during the training phase^42^. The learning rate, denoted as γ, utilized with this dataset had an initial value of 1 × 10^−3^ and was multiplied by a fixed multiplier of 0.1 after 60, 120, and 180 epochs. Data augmentation is a commonly used method to expand a training dataset^43^. For data augmentation, the training data were sampled by a random 24 × 24 crop from an image padded by four pixels on each side. Adam with an L2 weight decay parameter λ of 1 × 10^−4^ was used as the optimizer^44^. We trained the network with ten random seeds by He initialization^45^. After training, an average classification accuracy of 99.37% for the test data was obtained.

### SVHN dataset

For SVHN, the network is characterized as 20**C**5-40**C**5-40**C**5-40**C**5-100**C**3**-**100**C**3**-**100**C**3**-**100**C**3-**FC**500-**FC**10, where *n***C***m* represents *n* filters of size *m* × *m* and **FC***m* denotes a fully connected layer with *m* neurons. For all hidden layers, several nodes randomly dropped out with a probability of 0.5 during the training phase. Stochastic gradient descent as an optimizer was used with a learning rate γ of 3 × 10^−5^_,_ an L2 weight decay parameter λ of 0.9, and momentum of 0.5. After training, a classification accuracy of 95.11% was obtained for the test data.

### CIFAR-10 dataset

For the CIFAR-10 dataset, we implemented a network consisting solely of convolution layers^46^. In a CNN, max-pooling is the most successful technique for subsampling. Several methods capable of converting a max-pooling layer to a SNN have been reported. Typically, lateral inhibition can be used, but this method does not guarantee the selection of a node with the maximum firing rates^15^. Another approach is to add a control gate that allows only the node with the maximum firing rate to pass, but considering the hardware implementation, an extra circuit is required for the gating function, which is a disadvantage in terms of area and energy consumption^8^. Thus, in this work, subsampling was performed using strided convolution layers, and global average pooling (GAP) was applied to the output layer^46^. The network in this case was 96**C**3-96**C**3-96**C**3(2)-192**C**3-192**C**3-192**C**3(2)−192**C**3-192**C**1-10**C**1-GAP, where *n***C***m*(*s*) indicates *n* filters of size *m* × *m* with stride *s*, and dropout with a probability of 0.5 was applied only to the strided convolution layers. The learning rate γ was initially 1 × 10^−3^ and was multiplied by a fixed multiplier of 0.5 after 50, 100, and 200 epochs. The stochastic gradient descent (SGD) algorithm was used as the optimizer with momentum of 0.9 and a L2 weight decay parameter λ of 1 × 10^−4^. Data augmentation was used based on a random 32 × 32 crop from an image padded by four pixels on each side and with horizontal flipping. After training, an average classification accuracy of 91.70% was obtained for the test data with ten random initial weights.

### Autoencoder

In classification problems where the most frequently firing neuron of the output layer matters, a slight performance drop can occur if the I&F neuron parameters are not optimal. In order to examine the effect of the proposed methods on more general SNN inference, an autoencoder capable of image compression and decompression was trained, as this method is a neural network application in which the actual activation value itself is important. The encoding part of the autoencoder is expressed as 128**C**3-256**C**3(2)-128**C**3(2)-4**C**3 and was accordingly capable of extracting 256 features. The decoding part was 128**C**^**T**^3-256**C**^**T**^3(2)-128**C**^**T**^3(2)-3**C**^**T**^3, restoring the features to the original images, where *n***C**^**T**^*m*(*s*) represents *n* deconvolution filters of size *m* × *m* and with stride *s*. The learning rate γ was initially 1 × 10^−3^ and was multiplied by a fixed multiplier of 0.1 after 120, 240, and 360 epochs. CIFAR-10 was used as the training data, and a sample consisting of a 512 × 512 image was split into 256 patches for the test data. The Adam optimizer was used with an L1 regularization parameter λ of 1 × 10^−9^.

### ANN-to-SNN conversion

All of the aforementioned networks were converted to a SNN using a previously reported ANN-to-SNN conversion method^7–9,15^. The trained weights were normalized by data-based normalization in order to ensure that the ANN output activations match the capacity of the firing rates of the I&F neurons in the SNN^7^. The normalization factors can be determined as the maximum output activation or maximum weight which prevents a single weight from driving too much of the activation. For GAP, generally, it can be simply converted to a SNN by connecting pooling filters with a weight of $\frac{1}{filter\_size}$, but this can overly suppress the firing rates of the output layer. Like data-based normalization, the GAP weights can be divided by the maximum output activation, resulting in an increase of the firing rate to the maximum allowable firing rate of an I&F neuron.
